# Effects of Inertial Flywheel Training vs. Accentuated Eccentric Loading Training on Strength, Power, and Speed in Well-Trained Male College Sprinters

**DOI:** 10.3390/life14091081

**Published:** 2024-08-29

**Authors:** Zhongzhong Hu, Yuhang Liu, Keke Huang, Hao Huang, Yu Zhang, Xiaoyi Yuan

**Affiliations:** 1 School of Sports Science, Wenzhou Medical University, Wenzhou 325035, China; bsu2014hzz@163.com (Z.H.); huangkekerunning@163.com (K.H.); huanghaomedical@163.com (H.H.); wmu_zhangyu@163.com (Y.Z.); 2 China Athletics College, Beijing Sport University, Beijing 100084, China; 15031958573@163.com

**Keywords:** resistance training, flywheel, accentuated eccentric loading, performance

## Abstract

This study aimed to evaluate and compare the effects of inertial flywheel training and accentuated eccentric loading training on the neuromuscular performance of well-trained male college sprinters. Fourteen sprinters were recruited and randomly assigned to either the flywheel training (FWT, *n* = 7) group or the accentuated eccentric loading training (AELT, *n* = 7) group. The FWT group completed four sets of 2 + 7 repetitions of flywheel squats, whereas the AELT group performed four sets of seven repetitions of barbell squats (concentric/eccentric: 80%/120% 1RM). Both groups underwent an eight-week squat training program, with two sessions per week. A two-way repeated ANOVA analysis was used to find differences between the two groups and between the two testing times (pre-test vs. post-test). The results indicated significant improvements in all measured variables for the FWT group: 1RM (5.0%, ES = 1.28), CMJ (13.3%, ES = 5.42), SJ (6.0%, ES = 2.94), EUR (6.5%, ES = 4.42), SLJ (2.9%, ES = 1.77), and 30 m sprint (−3.4%, ES = −2.80); and for the AELT group: 1RM (6.3%, ES = 2.53), CMJ (7.4%, ES = 3.44), SJ (6.4%, ES = 2.21), SLJ (2.2%, ES = 1.20), and 30 m sprint (−3.0%, ES = −1.84), with the exception of EUR (0.9%, ES = 0.63, *p* = 0.134), showing no significant difference. In addition, no significant interaction effects between group and time were observed for 1RM back squat, SJ, SLJ, and 30 m sprint (*p* > 0.05). Conversely, a significant interaction effect between group and time was observed for both CMJ and EUR (*p* < 0.001); post hoc analysis revealed that the improvements in CMJ and EUR were significantly greater in the FWT group compared to the AELT group (*p* < 0.001). These findings indicate that both FWT and AELT are effective at enhancing lower-body strength, power, and speed in well-trained male college sprinters, with FWT being particularly more effective in promoting elastic energy storage and the full utilization of the stretch–shortening cycle.

## 1. Introduction

A sprint is a track and field event that requires running over a short distance at maximum speed [1], highlighting the athlete’s explosive power, acceleration, optimal running technique, and neuromuscular system’s ability to rapidly alternate between states of excitation and inhibition [2,3,4,5,6]. Modern sprint training systems place a significant emphasis on strength training [7], as increasing muscle strength and power can greatly enhance mechanical efficiency, muscular coordination, motor unit recruitment patterns, and muscle and tendon stiffness [7,8]. These improvements are crucial for building specific strength in key muscle groups essential for competitive success. Running at maximal speed poses high neurophysiological and biomechanical demands on the sprinter’s body [9,10]. Eccentric strength may directly enhance lower-limb stiffness and reactive strength by preventing excessive muscle lengthening under high stretch loads, and indirectly by increasing force production during a subsequent quasi-isometric action through residual force enhancement [11]. Additionally, prolonged and intense eccentric strain from overload may preferentially recruit high-threshold motor units [12]. This recruitment enables the generation of greater maximal force with lower metabolic cost for the same amount of work, maximizing muscle strength gains, regional hypertrophy, and remodeling of muscle architecture [13]. Several studies have shown that eccentric training can accelerate or optimize improvements in maximal muscular strength, power development, optimal muscle length for strength gains, and coordination during eccentric movements [14,15,16,17]. Therefore, the systematic inclusion of eccentric-based training protocols in strength and conditioning programs can enhance sprinter performance and help prevent injuries.

Numerous studies have confirmed that the Nordic hamstring exercise effectively improves the eccentric hamstring strength of sprinters [18,19,20]. However, during sprinting, particularly at maximum speed, the hamstring muscles must generate significant eccentric force in a short amount of time to decelerate knee extension, resist the powerful concentric contraction of the quadriceps [21], and capitalize on reflex potentiation [22]. This maximizes the utilization of elastic structures within the stretch–shortening cycle (SSC) [23]. In other words, the slow movement demands of the Nordic hamstring exercise do not align with the hamstring muscle action patterns required during high-speed running. Consequently, trainers and practitioners are exploring alternative training methods to enhance the strength and force production of eccentric muscle actions and to increase the specificity and efficiency of the SSC. Eccentric overload training (EOT) refers to the training condition where the load intensity applied during the eccentric phase exceeds that of the concentric phase. This approach allows the body to withstand greater loads, disrupt conventional neural adaptations, and activate a larger number of motor units [24,25,26]. EOT can reduce muscle resistance at the weakest point of motion, increase resistance where strength is greater, and more closely match human strength curves, allowing muscles to operate over a wider range [27,28]. Various methods are employed to achieve eccentric overload, including manual force application, elastic bands, computer-driven devices, weight releasers, and inertial flywheel devices. Among these, flywheels and weight releasers are the most common due to their minimal disruption to exercise mechanics [29].

Specifically, inertial flywheel devices, which harness the inertia of a rotating wheel and the subsequent stored kinetic energy, offer a higher eccentric load compared to traditional weight-training methods [30]. Eccentric overload training using flywheel devices has been shown to be effective in improving jumping and linear sprinting performance in soccer players [31]. Similarly, Murton et al. reported that flywheel and traditional resistance training are equally effective in enhancing lower-body strength and power in male academy rugby union players [32]. Flywheel training (FWT) leads to a significant decrease in muscle oxygen saturation and a longer reoxygenation time, thereby imposing greater physiological stress than traditional strength training [33]. The primary reason FWT induces neural adaptations, such as increased peak power output, muscle cross-sectional area, and tendon stiffness, is the improvement in motor unit recruitment, rate coding (firing frequency), synchronous motor unit activity, and reduced neuromuscular inhibition [12,34]. It is important to note that, despite the advantages and positive aspects discussed earlier, there are some disadvantages associated with the use of inertial flywheel devices, including high costs and difficulties in precisely controlling and prescribing the adequate training load [35,36]. These challenges may hinder or, at the very least, reduce the likelihood of coaches incorporating isoinertial resistance training into their daily practices. The currently popular accentuated eccentric loading training (AELT), which uses only a barbell and weight releasers—adjustable hooks that detach at the lift’s lowest point to achieve accentuated eccentric overload—has also been shown to improve maximum strength, power, and sprint ability [29,37,38,39,40]. The primary reason is that AELT can significantly increase the cross-sectional area of type IIx muscle fibers and promote shifts towards faster myosin heavy chain isoforms, which are associated with enhancements in force and power production [34,38]. AELT not only allows for precise quantification of training load, but also makes it more affordable; however, it often requires third-party assistance. FWT and AELT produce eccentric overload in different ways, each with its own advantages and disadvantages. This study represents the first attempt to compare the effects of FWT and AELT using weight releasers on the neuromuscular performance of sprinters.

## 2. Materials and Methods

### 2.1. Experimental Approach to the Problem

Resistance-trained academy sprinters were recruited to elucidate the effects of AELT vs. FWT on strength, power, and speed. Subjects were randomly assigned to complete either AELT or FWT protocols within their resistance-training program based on a computer-generated sequence (www.randomizer.org, accessed on 15 April 2023). The primary difference between groups was the way to produce eccentric overload. All other elements of the resistance-training program such as exercise selection, sets, repetitions, and rest intervals and frequency were matched between groups. In addition, all subjects were recruited from the same academy program; therefore, the weekly schedule and training load were approximately equivalent across all subjects for the duration of the study. Both AEL and TRT groups completed two training sessions weekly for eight weeks. Dependent variables including muscle strength, power, and speed were assessed before and after the intervention.

### 2.2. Subjects

Our study employed a randomized two-group design with repeated measures. We calculated the required sample size using G*Power 3.1.9.7 Software (Düsseldorf, Germany), aiming for a power of 90% (1 − β = 0.90), an alpha error rate of 0.05, and an effect size of 0.95. This effect size was derived from a preliminary experiment on the countermovement jump, a reliable measure of lower-limb power in sprinters [5]. Accordingly, a minimum of 12 subjects was necessary. We successfully enrolled 14 elite academy sprinters (age = 23.5 ± 0.5 years; height = 181.0 ± 2.3 cm; body mass = 77.0 ± 2.6 kg; back squat 1RM = 148.2 ± 10.7 kg; back squat to 1RM ratio = 1.92 ± 0.28, Table 1). To minimize potential biases, subjects were required to meet specific inclusion criteria: (1) absence of lower-extremity musculoskeletal injuries in the past year; (2) well-trained collegiate male sprinters with a minimum of 4 years of training experience; (3) consistent engagement in lower-body resistance training at least twice per week for the six months preceding the study; (4) ability to squat at least 1.5 times their body weight to a depth where the thigh is parallel to the ground. During the program, their typical training volume included five sessions, each lasting 90 min. The weekly training regimen was structured as follows: two sessions focused on speed training, one session dedicated to specific physical fitness exercises and aerobic recovery, and two sessions concentrated on strength training using weight releasers or flywheel devices. Although the participants were highly experienced with free-weight and weight-stack machines, none of them had prior experience with weight releasers or flywheel devices during back squat exercises. The Research Ethics Committee of Beijing Sport University approved the study protocol (Registration number 2023215H), ensuring all procedures adhered to the Declaration of Helsinki standards. Prior to participation, all participants provided written informed consent, acknowledging the study’s nature, benefits, and risks.

### 2.3. Procedures

**Figure 1:**
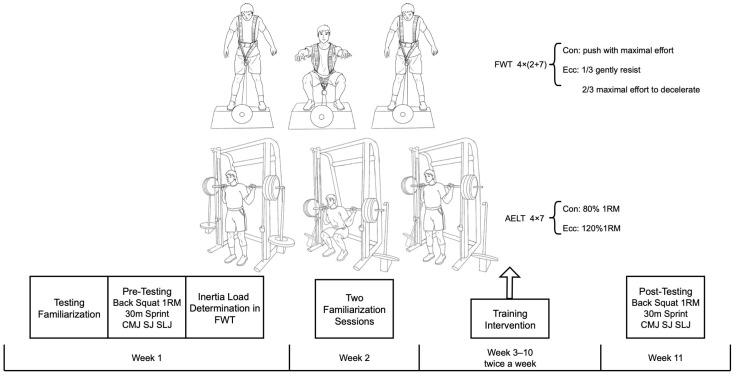
Schematic diagram of the experimental process.

The experimental process of this study is depicted in Figure 1. Spanning 11 weeks, subjects were advised to refrain from any additional resistance training throughout the study period. The initial week comprised four sessions. The first session was dedicated to familiarizing subjects with the testing procedures for strength, sprinting, and jumping abilities. The second session involved administering back squat 1RM and 30 m linear sprint tests, while the third session focused on countermovement jump (CMJ), squat jump (SJ), and standing long jump (SLJ) tests. Before each test, a standardized warm-up routine was performed, which included 3 min of light jogging, 5 min of dynamic stretching, and 5 min of lower-body strength exercises, such as multi-directional lunges, bodyweight squats, and squat jumps. A rest period of three minutes was allotted between the final practice trial and the commencement of the tests. Subjects performed two practice trials for each test, exerting maximum effort, with at least two minutes of rest between trials. In the fourth session, the flywheel’s moment of inertia and the mass of discs for subjects in the FWT group were adjusted based on the mean movement velocity during the concentric phase. The following week included two familiarization sessions to ensure subjects fully comprehended the proper techniques and acclimated to the use of flywheel or weight releasers, aiming to optimize training adaptations. The intervention spanned eight weeks, with the FWT and AELT groups undergoing training sessions twice weekly, maintaining a recovery period of at least 48 h between sessions. Post-training assessments were conducted in two sessions: the first evaluated back squat 1RM and 30 m sprint, and the second measured CMJ, SJ, and SLJ performances. Measurements and data analysis were carried out at the indoor track and field and the scientific research facility of Beijing Sport University.

### 2.4. Inertial Load Determination

The experimental groups (FWT and AELT) underwent identical training regimens, maintaining the same number of sessions, repetitions, sets, and rest intervals to ensure equivolume training protocols. Traditionally, the intensity of resistance training using gravity-dependent devices has been prescribed as a percentage of the maximum strength (1RM). However, with flywheel technology, determining the 1RM is impractical due to the absence of a maximal load limit. Despite peak power frequently serving as the primary parameter [35] to assess flywheel exercise intensity (e.g., during squats), recent studies advocate for velocity monitoring (mean and peak) over power as a more accurate measure [41,42]. Consequently, we adopted the mean concentric velocity as the criterion for setting the inertial load on the flywheel. Subjects were advised against shoulder shrugging at full hip extension and disallowed ankle extension. They were instructed to execute the concentric phase rapidly and to modulate the inertial force in the initial third of the eccentric action, subsequently exerting maximal effort to halt the movement at the motion’s end range [43]. Subjects performed 2 + 7 repetitions of the flywheel squat; it should be noted that the first two repetitions, aimed at ‘increasing momentum’, were not included in the data analysis [40]. A linear transducer (GymAware Power Tool; Kinetic Performance Technologies, Canberra, Australia) was affixed to the chest strap during flywheel squats or positioned 50 mm away from the left hand for barbell squats. The GymAware system is renowned for its accuracy. It uses an extremely accurate LPT linear displacement sensor with a sampling rate of 100 Hz, a distance resolution of 0.8 mm, and an angle sensor resolution of 0.1 degrees, which can avoid the impact of non-vertical movements. It has served as the benchmark for velocity monitoring devices’ validation in numerous studies [44]. Immediate velocity performance feedback was provided post-repetition to motivate subjects to maintain maximal velocity throughout. Rest intervals between sets were standardized to three minutes. The inertial load for flywheel squats was adjusted based on the subjects’ mean concentric velocity during 7 repetitions of 80% 1RM barbell squat. Prior research has documented that the mean concentric velocities while squatting with a load equivalent to 80% 1RM were 0.55 m/s [45] and 0.62 m/s [46]. Our findings align closely with these figures, recording a similar velocity of 0.58 ± 0.04 m/s. And subjects in the FWT group used an inertial load of 0.05~0.112 kg/m^2^, which was consistent with Brien et al. [47] reporting that moderate or large inertial loads maximized eccentric overload. 

### 2.5. Training Program 

Subjects engaged in resistance-training protocols for eight consecutive weeks, with sessions held twice weekly, spaced at least 48 h apart. The FWT group performed squats using a flywheel device (Desmotec, D.11 Full, Biella, Italy), executing 4 sets of 2 + 7 repetitions (the first and second repetitions were used to increase the velocity of the weighted disc and were excluded from the data analysis). Conversely, the AELT group conducted squats on a Smith machine equipped with weight releasers, completing 4 sets of 7 repetitions. Both groups took a 3 min rest between sets. FWT subjects were instructed to exert maximal effort throughout the entire concentric phase, from 70° knee flexion to near full extension. Initially, they were to gently resist during the first third of the eccentric phase, then apply maximal braking force to halt the movement at approximately 70° knee flexion [48]. The AELT group performed back squats using barbells equipped with weight releasers (Titan Fitness, Memphis, TN, USA) to induce eccentric overload, setting the concentric load at 80% of their 1RM and the eccentric load at 120% of their 1RM, as this approach was based on prior research [28,49] that training with high loads can be more effective in improving muscle strength and recruitment than low-load training. The height of the weight releasers was adjusted to each participant’s lowest squat depth, ensuring disengagement just before the barbell reached the lowest squat position. AELT subjects received strong verbal encouragement to perform the concentric phase explosively in each session. To achieve eccentric overload, two training supervisors reloaded the weight releasers onto the barbell between each repetition.

Back Squat One-Repetition Maximum. Dynamic strength was assessed using a well-established one-repetition maximum (1RM) back squat protocol [50]. Subjects achieved their 1RM within 3 to 4 maximal efforts, following a standardized warm-up tailored to their self-reported 1RM back squat. Starting with feet shoulder-width apart and the barbell positioned on the upper back at shoulder level, with knees and hips fully extended, subjects were directed to lower themselves until their thighs were parallel to the floor, then rise back to a standing position at maximum speed. The squat depth was verified by two supervisors positioned to the side of the power rack. Details such as foot placement, rack height, and safety bar adjustments were documented for consistency in future sessions. The successfully recorded 1RM was then utilized to determine the load for experimental conditions.

Countermovement Jump and Squat Jump. The heights of the countermovement jump (CMJ) and squat jump (SJ) were measured using a Chronojump contact mat (Chronojump, Barcelona, Spain), which has demonstrated excellent reliability (ICC 0.998 for the SJ and 0.997 for the CMJ) compared to the Force Platform [51]. Subjects executed two attempts of each jump type, aiming for maximum height with hands placed on the hips. For the SJ, subjects began from a static position with knees bent at 90°, eliminating the eccentric phase. The CMJ was initiated from a standing position, seamlessly transitioning between the eccentric and concentric phases without pausing, and the knee flexion angle was chosen by the participant. A 30 s active recovery period was provided between attempts. The highest values recorded for both SJ and CMJ were used for subsequent analysis. The eccentric utilization ratio (EUR) was calculated as CMJ divided by SJ.

Standing Long Jump. Each participant positioned themselves on the starting line, with legs aligned parallel and feet placed shoulder-width apart. They then bent their knees and positioned their arms behind their body. Following this, they executed a forceful leg extension, propelled their arms forward, and performed a maximal jump for distance. The jump distance was measured in centimeters by the same individual. Subjects completed two attempts of the standing long jump, with 1 min rest intervals between jumps. The best attempt was documented for statistical analysis.

30 m Sprint. The 30 m sprint time was accurately measured using timing gates with error-correction processing algorithms (Smartspeed pro, Fusion sport, Milton, Australia). This test involved a maximal effort sprint in a straight line across two timing gates spaced 30 m apart, starting from a standing position. Subjects positioned themselves 30 cm behind the starting photocell, which was 80 cm above the ground, placing their preferred foot forward with the toe touching the line marked on the ground. The timing commenced as subjects activated the electronic sensors and ceased as they passed through the sensor plane again. Each participant undertook two trials, interspersed with 3 min active recovery periods. All evaluations were conducted on an indoor plastic track, and subjects were required to wear running shoes. The quickest trial was selected for statistical analysis.

**Figure 2:**
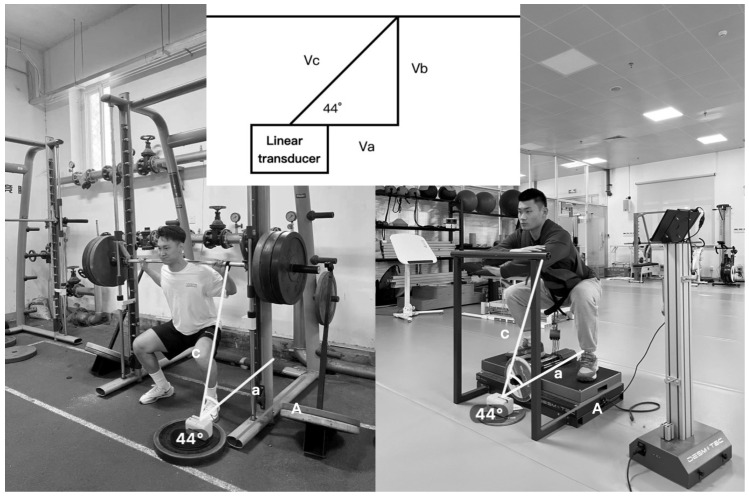
The linear transducer showing a deviation of 44° from the vertical during a lift.

Mechanical Variables. Measures of mean concentric power (MCP) and velocity (MCV) were collected in the execution of all repetitions by a valid and reliable linear position transducer (LPT) (GymAware, Kinetic Performance Technology, Canberra, Australia). Through the LPT, velocity data were calculated from the displacement of the bar or strap relative to time, and the power was calculated by the multiplication of force by velocity. To ensure the data’s authenticity and reliability, we confirmed the position of the linear sensor before performing both the flywheel squat and Smith machine squat, verifying that the angles were identical. The angle between the cable and the ground, consistently measured by Dartfish 2022 pro software (Fribourg, Switzerland) at 44 degrees, is illustrated in Figure 2 Based on the application of the Pythagorean theorem in previous research [46], the velocity of the cable (Vc), as recorded by the linear sensor, can be utilized to determine the vertical velocity (Vb) during barbell lifting. Specifically, Vb is calculated as Vc multiplied by the sine of α, which is Vb = Vc × sin (44°) = 0.695 Vc. Data obtained from GymAware were transmitted via Bluetooth to a tablet (iPad, Apple Inc., Cupertino, CA, USA) using the GymAware v2.1.1 app. We chose the last training session to compare the mechanical effects of each training protocol, which were assessed by perceptual maintenance and decline of velocity and power with the following equation, as proposed by Tufano et al. [52]:

Percent decline = [(repetition_lowest_ − repetition_highest_)/repetition_highest_] × 100

Maintenance = 100 − [(repetition_highest_ − repetition_mean_)/repetition_highest_ × 100]

### 2.6. Statistical Analysis

Statistical analyses were conducted using IBM SPSS statistics software (version 26.0, IBM, Chicago, IL, USA). The results are expressed as the mean ± standard deviation. The Shapiro–Wilk test and Levene’s test were utilized to assess data normality and equality of variances, respectively. A two-way repeated-measures ANOVA was applied to examine the effects of the group (FWT vs. AELT) and time (Pre vs. Post). Additionally, within-group comparisons of training variables were performed using paired *t*-tests, and an independent sample *t*-test was used in between-group comparisons of mechanical variables. The effect size (ES) for training was calculated for paired variables, following Cohen’s classification [53], where ES of 0.2, 0.5, 0.8, and 1.3 indicate small, moderate, large, and very large effects, respectively. The threshold for statistical significance was set at *p* < 0.05.

## 3. Results

Subjects from both groups successfully completed all the designated training sessions. Table 2 presents the results for the 1RM back squat, vertical jump, eccentric utilization ratio, standing long jump, and 30 m sprint time.

### 3.1. Back Squat 1RM

No significant main effects for group (F_group = 1.263; *p* = 0.283) or the interaction between group and time (F_group × time = 3.698; *p* = 0.079) were observed on 1RM back squat values. However, a significant main effect for time was noted (F_time = 262.717; *p* < 0.001). Post hoc analysis revealed a significant increase in 1RM among both FWT and AELT subjects following the training intervention, compared to their baseline values (FWT: *p* < 0.001, ES = 1.28; AELT: *p* < 0.001, ES = 2.53). Furthermore, 1RM increased by 5.0% in the FWT group and by 6.3% in the AELT group post-intervention. 

### 3.2. Vertical Jump and Eccentric Utilization Ratio

Significant main effects of group, time, and the interaction between group and time on CMJ height were observed (F_group = 11.181, *p* = 0.006; F_time = 428.404, *p* < 0.001; F_group × time = 34.833, *p* < 0.001, respectively). Post hoc analysis demonstrated a significant increase in CMJ height for both FWT and AELT subjects post-training compared to the baseline (FWT: *p* < 0.001, ES = 5.42; AELT: *p* < 0.001, ES = 3.44). However, FWT subjects achieved significantly higher CMJ height than AELT subjects post-training (*p* < 0.001). Additionally, CMJ height in the FWT group increased by 13.3%, while the AELT group experienced a 7.4% increase post-intervention. No significant main effects for group (F_group = 1.416; *p* = 0.257) or the interaction between group and time (F_group × time = 0.994; *p* = 0.339) were observed for SJ height. However, a significant main effect for time was noted (F_time = 1956.791; *p* < 0.001). Post hoc analysis revealed a significant increase in SJ height among both FWT and AELT subjects following the training intervention, compared to their baseline values (FWT: *p* < 0.001, ES = 2.94; AELT: *p* < 0.001, ES = 2.21). Furthermore, SJ height increased by 6.0% in the FWT group, and by 6.4% in the AELT group post-intervention.

Significant main effects of group, time, and the interaction between group and time on EUR values were observed (F_group = 7.317, *p* = 0.0019; F_time = 63.473, *p* < 0.001; F_group × time = 36.528, *p* < 0.001, respectively). Post hoc analysis demonstrated a significant increase in EUR values for FWT subjects post-training compared to the baseline (*p* < 0.001, ES = 4.42). In contrast, the EUR values of AELT subjects did not show a significant increase post-training compared to the baseline (*p* = 0.199, ES = 0.63). Additionally, post hoc analysis revealed that the EUR values after the training period were significantly higher in the FWT group compared to the AELT group (*p* < 0.001). EUR values in the FWT group increased by 6.5%, while the AELT group experienced a 0.9% increase post-intervention.

### 3.3. Standing Long Jump

No significant main effects for group (F_group = 1.023; *p* = 0.332) or the interaction between group and time (F_group × time = 2.557; *p* = 0.136) were observed for SLJ distance. However, a significant main effect for time was noted (F_time = 130.435; *p* < 0.001). Post hoc analysis revealed a significant increase in SLJ distance among both FWT and AELT subjects following the training intervention, compared to their baseline values (FWT: *p* < 0.001, ES = 1.77; AELT: *p* < 0.001, ES = 1.20). Furthermore, SLJ distance increased by 2.9% in the FWT group, while the AELT group experienced a 2.2% increase post-intervention.

### 3.4. 30 m Sprint

No significant main effects for group (F_group = 0.356; *p* = 0.562) or the interaction between group and time (F_group × time = 1.069; *p* = 0.321) were observed for S30 time. However, a significant main effect for time was noted (F_time = 251.406; *p* < 0.001). Post hoc analysis revealed a significant increase in S30 time among both FWT and AELT subjects following the training intervention, compared to their baseline values (FWT: *p* < 0.001, ES = −2.80; AELT: *p* < 0.001, ES = −1.84). Furthermore, S30 decreased by 3.4% in the FWT group, while the AELT group experienced a 3.0% decrease post-intervention.

### 3.5. Mechanical Variables

**Figure 3:**
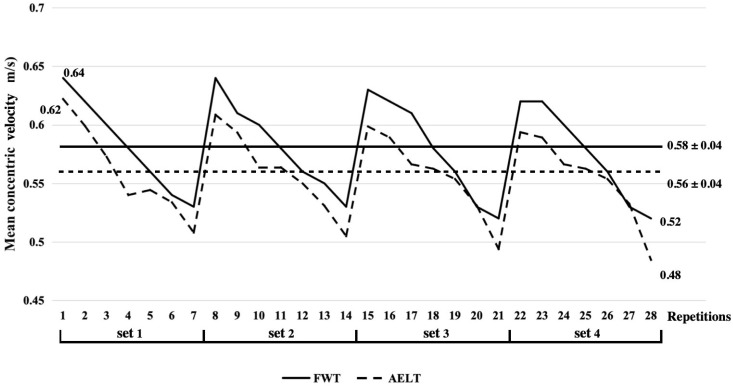
Mean concentric velocity of each repetition flywheel training and accentuated eccentric loading training during the last training session.

**Figure 4:**
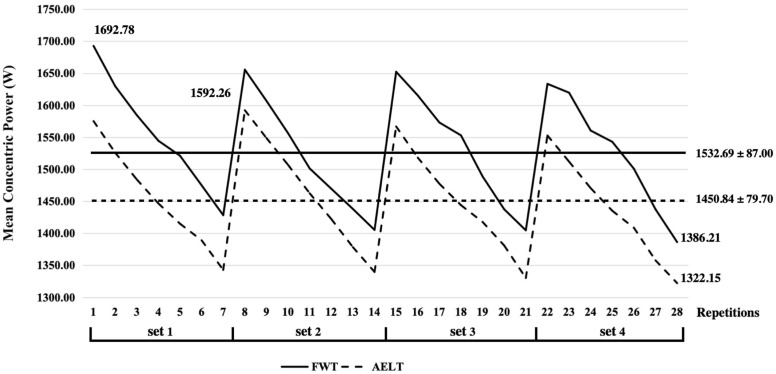
Mean concentric power of each repetition flywheel training and accentuated eccentric loading training during the last training session.

The independent sample *t*-test did not detect significant differences between FWT and AELT regarding the mean concentric velocity (0.58 ± 0.04 m/s and 0.56 ± 0.04 m/s for FWT and AELT, respectively; *p* = 0.411; Figure 3) and peak concentric velocity (1.06 ± 0.13 m/s and 1.03 ± 0.12 m/s for FWT and AELT, respectively; *p* = 0.852). Similarly, differences between groups regarding mean concentric velocity decline (−20.3 ± 7.7% and −22.6± 6.9% for FWT and AELT, respectively; *p* = 0.330) and maintenance (90.6 ± 3.4% and 90.3 ± 3.1% for FWT and AELT, respectively; *p* = 0.476) were not significant. However, mean eccentric velocity was significantly higher in FWT (0.62 ± 0.06 m/s) versus AELT (0.50 ± 0.04 m/s; *p* < 0.001). There were significant differences between FWT and AELT concerning the mean concentric power (1532.69 ± 87.00 W and 1450.84 ± 79.70 W for FWT and AELT, respectively; *p* = 0.03; Figure 4) and peak concentric power (2325.32 ± 295.26 W and 2115.66 ± 243.80 W for FWT and AELT, respectively; *p* = 0.02). However, differences between groups regarding mean concentric power decline (−18.1 ± 5.5% and −17.0 ± 6.6% for FWT and AELT, respectively; *p* = 0.852) and maintenance (90.5 ± 2.9% and 91.1 ± 3.3% for FWT and AELT, respectively; *p* = 0.757) were not significant.

## 4. Discussion

This study aimed to explore the impact of an eight-week FWT versus AELT on the neuromuscular performance of well-trained male college sprinters. Our findings suggest that both FWT and AELT significantly enhanced lower-body strength, power, and linear sprint capabilities. Notably, the countermovement jump (CMJ) and eccentric utilization ratio (EUR) observed in FWT subjects were superior to those in the AELT group. 

The mean concentric velocity (MCV) in the FWT and AELT groups experienced a notable decline between repetitions. However, after a 3 min rest period, the initial repetition of the subsequent set could achieve a relatively high velocity. It is evident that the MCV in each repetition for the AELT group was consistently lower than that observed in the FWT group. It is important to note mean concentric velocity maintenance—a variable highlighting the comparison between the highest velocity of a session and the session’s mean velocity. Our findings indicate no significant difference in MCV maintenance between the two groups. The velocity–time profile observed in the FWT group aligns with known characteristics of flywheel training, where the inertial force of the flywheel offers unrestricted resistance from the onset, promoting maximum or near-maximum muscle activation [54]. As repetitions increase, so does fatigue accumulation, leading to a decrease in mean velocity. Additional effort needed to slowly unrack heavy weights (120% of 1RM) during AELT may reduce metabolite clearance in working muscles, potentially impairing the maintenance of mechanical performance.

The mean concentric power (MCP) in the FWT and AELT groups exhibited a significant decline between repetitions. Simultaneously, it is observed that the MCP for each repetition in the AELT group was lower than in the FWT group. However, following a 3 min rest period, the first repetition of the subsequent set was able to attain a relatively high power level. This pattern suggests that longer rest intervals might facilitate better restoration of energy substrates. However, this interpretation remains speculative, as the restoration of energy substrates was not directly assessed in this study.

Our study’s findings reveal no significant differences in maximal squat strength gains between the two training protocols. This observation aligns with our previous statement regarding the absence of any difference in mean and peak concentric velocity between the two groups. We observed a notable 5% increase in 1RM squat strength after eight weeks (two sessions per week) of FWT, aligning with Coratella et al. [55], who reported a 7% enhancement in 1RM following an identical period of FWT. The scientific literature acknowledges that FWT induces various morphological and neural adaptations, including enhanced motor unit recruitment, increased firing frequency [56], improved synchronization of motor unit activity [12], and reduced neuromuscular inhibition [34], all of which are critical for strength development. To elicit the optimal training response, subjects are required to concentrate on generating (near) maximal effort in each flywheel squat, as well as on the timing and technique of the braking force during the eccentric phase. This approach ensures (near) maximal muscle activation and enhances the workout’s intensity. Maroto-Izquierdo et al. [49] found significant strength improvements following ten weeks of eccentric overload training with supramaximal loads (120% 1RM). Similarly, Walker found that AELT resulted in greater increases in maximum force production, work capacity, and muscle activation, but did not lead to muscle hypertrophy in strength-trained individuals [38]. A potential explanation for the observed increase in maximal strength could be the enhanced neural stimulation of the muscle, attributed to the more substantial stretch of intrafusal muscle fibers (muscle spindles) under increased eccentric loads. This stretch prompts the intrafusal fibers to activate their associated γ motor neurons, which in turn signal the brain to either recruit more α motor neurons or to accelerate their firing rate. Consequently, this leads to a more forceful contraction of the extrafusal muscle fibers, as described by Dietz et al. [57]. Essentially, applying a heavier-loaded eccentric contraction neurologically primes the brain for a more potent concentric contraction, effectively “tricking” it into preparing for increased demands.

Our results demonstrated significantly greater enhancements in the countermovement jump (CMJ) and squat jump (SJ) across both groups, with FWT showing more pronounced improvements in the CMJ and EUR. These findings align with previous research [55,58,59,60]. Eccentric overload training, whether utilizing flywheel devices or weight releasers, has shown significant increases in type IIx fibers and shifts towards faster myosin heavy chain isoforms, leading to enhanced force and power production [29,61]. FWT has been shown to acutely enhance the rate of muscle lengthening, as evidenced in previous studies [62,63]. This enhancement may lead to temporary storage of additional elastic energy, subsequently available for use during concentric muscle actions. The rationale is based on the higher force generation capabilities and selective recruitment of high-threshold motor units during eccentric muscle actions, which can potentially elicit neuromuscular responses that lead to the desired adaptations [64,65]. Furthermore, the rapid transition between the eccentric and concentric phases during the flywheel squat enhances the stretch of the musculotendinous complex, thereby triggering a myotatic reflex that amplifies the subsequent concentric contraction, as demonstrated by Pecci et al. [66]. A comparison of mean eccentric velocity between groups revealed that the use of weight releasers induces trainers to adopt slower eccentric pacing strategies for managing supramaximal loads, which could reduce the full potential utilization of the stretch–shortening cycle (SSC) [65]. Regarding concentric power output, for both mean and peak power, the FWT group exhibited higher values compared to the AELT group. This discrepancy is likely due to the extended amortization phase in AELT, which may restrict the utilization of the SSC for concentric potentiation [67]. EUR has been established as a reliable indicator of SSC performance across various sports and training stages [68,69]. The present study corroborates this perspective, demonstrating that FWT significantly enhances EUR and CMJ performance in comparison to AELT. Essentially, an improvement in EUR attributable to FWT indicates superior elastic energy storage and utilization within the SSC, which contributes to increased CMJ height [70].

Significant improvements in 30 m sprint times were observed in both groups (FWT: 3.4%, AELT: 3.0%). According to the above research results for 1RM and CMJ, it can be seen that these two have a positive effect on increasing sprint speed. Indeed, it has been consistently demonstrated that improvements in back squat strength positively transfer to sprinting speed [2]. Moreover, the storage and return of energy within the elastic structures of the lower limb play an increasingly important role at higher sprinting speeds up to maximum velocity [4,23,71]. The outcomes in our study align with Petre et al. [72], who reported a 2.4% enhancement in sprint performance after 6–10 weeks of FWT (1–3 sessions per week) among well-trained athletes. Similarly, Cabanillas et al. [73] found that flywheel squat training positively influenced force production in the horizontal vector, leading to improved 30 m sprint times. Douglas et al. [50] also noted that AELT is an effective strategy for boosting sprint ability in rugby players. Previous research [74] has validated the efficacy of eccentric training protocols in increasing tendon stiffness, which significantly enhances sprint performance [23]. Overall, our study provides strong evidence supporting the notion that eccentrically emphasized resistance exercise can potentially contribute to superior neuromuscular adaptation [75], such as improved sprint ability.

Our findings demonstrate that both FWT and AELT are beneficial for enhancing standing long jump (SLJ) performance, with increases of 2.9% for FWT and 2.2% for AELT, but no significant differences in the two training modalities were detected. Increasing the maximum strength of the lower limbs has been conclusively shown to enhance their explosive power [76]. Correspondingly, the aforementioned study reported improvements in maximum squat strength for both experimental groups, which were directly linked to enhancements in the standing broad jump performance. Previous research has shown that flywheel squat exercises have acute enhancement in SLJ ability [77,78], while our study reveals a long-term positive impact of FWT on SLJ performance. Previous studies have confirmed that flywheel squat training significantly contributes to improvements in horizontal jumping, acceleration, and linear sprinting [73,79], all crucial for sprinters. The impact of accentuated eccentric load (AEL) on the SLJ has not been previously investigated. It is likely that AEL training (AELT) enhances SLJ performance due to increased muscle lengthening rates and the potentiation of concentric actions through the activation of higher threshold motor units, as well as the preservation of elastic energy [80]. 

The study has several limitations that warrant attention. First, the findings, derived from well-trained male college sprinters, may only be generalizable to comparable cohorts and competitive levels. Second, a methodological consideration needs to be acknowledged. Despite efforts to standardize training volume and intensity across groups, achieving an exactly equal workload proved challenging. Although it is feasible to quantify the training volume of accentuated eccentric loading, such quantification is not viable with flywheel devices. Future studies should conduct multi-center, large-sample, and long-term randomized controlled trials to provide more reliable evidence-based support for resistance strength training in sprinters. Additionally, it is crucial to explore the impact of diverse tempo strategies in FWT, including variations in movement velocity during the concentric and eccentric phases, and the eccentric-to-concentric load ratio in AELT on training outcomes. Another important research direction is examining the distinct effects of training with different equipment, such as vertical flywheel devices, horizontal flywheel devices, and seated leg curl flywheel devices.

## 5. Conclusions

The results of this experimental training study indicate that eight weeks of either FWT or AELT significantly enhanced back squat strength and linear sprint performance in well-trained male college sprinters. Additionally, both FWT and AELT led to significant improvements in jumping abilities, with FWT being more effective in promoting elastic energy storage and full utilization of the stretch–shortening cycle. Consequently, both methods are valuable for integrating into training regimens to facilitate strength and power adaptations. It is recommended to incorporate FWT and AELT into sports periodization to diversify training stimuli for well-trained athletes.

## Figures and Tables

**Table 1:** Basic characteristics of sprinters included in the analysis.

	FWT Group (*n* = 7)	AELT Group (*n* = 7)	Difference (*p*-Value)
Age (year)	23.2 ± 0.6	23.8 ± 0.6	0.749
Height (cm)	181.5 ± 3.7	180.6 ± 3.2	0.654
Body weight (kg)	77.4 ± 3.6	76.7 ± 2.4	0.833
Training age (year)	5.1 ± 0.9	5.3 ± 0.6	0.784
1RM back squat (kg)	147.3 ± 9.4	149.1 ± 11.5	0.481
PB time for 100 m (s)	10.87 ± 0.06	10.90 ± 0.06	0.247

Values are mean ± SD and *p*-value of the differences. FWT = flywheel training; AELT = accentuated eccentric loading training; 1RM = one-repetition maximum; PB = personal best.

**Table 2:** Between-group and within-group differences in selected variables with % of improvement and Cohen’s effect size (d).

	FWT (*n* = 7)				AELT (*n* = 7)				*p* (Time)	*p* (Group)	*p* (Time × Group)
Variables	Pre	Post	Δ%	Cohen’s d	Pre	Post	Δ%	Cohen’s d			
1RM (kg)	147.29 ± 15.41	154.71 ± 1 6.10 **	5.0	1.28	149.14 ± 14.06	158.57 ± 13.36 **	6.3	2.53	<0.001	0.283	0.079
CMJ (cm)	50.52 ± 2.19	57.22 ± 2.28 **##	13.3	5.42	50.10 ± 2.86	53.83 ± 2.27 **	7.4	3.44	<0.001	0.006	<0.001
SJ (cm)	47.22 ± 1.71	50.07 ± 1.93 **	6.0	2.94	46.41 ± 1.36	49.39 ± 1.34 **	6.4	2.21	<0.001	0.257	0.339
EUR	1.07 ± 0.02	1.14 ± 0.01 **##	6.5	4.42	1.08 ± 0.02	1.09 ± 0.01	0.9	0.63	<0.001	0.019	<0.001
SLJ (m)	2.73 ± 0.08	2.81 ± 0.05 **	2.9	1.77	2.71 ± 0.07	2.77 ± 0.05 **	2.2	1.20	<0.001	0.332	0.136
S30 (s)	4.09 ± 0.05	3.95 ± 0.05 **	−3.4	−2.80	4.06 ± 0.07	3.94 ± 0.06 **	−3.0	−1.84	<0.001	0.562	0.321

FWT = flywheel training; AELT = accentuated eccentric loading training. One-repetition maximum = 1RM, countermovement jump = CMJ, squat jump = SJ, eccentric utilization ratio (EUR) = CMJ/SJ, standing long jump = SLJ, linear sprint 30 m = S30. Data are presented as mean ± SD, significant Cohen’s d, and *p*-values. Δ% = percentage of improvement, ** = within-group statistical significance (*p* < 0.01), ## = between-group statistical significance (*p* < 0.01).

## Data Availability

The original contributions presented in the study are included in the article, further inquiries can be directed to the corresponding author.
